# Clinical performance of resin-matrix ceramic partial coverage restorations: a systematic review

**DOI:** 10.1007/s00784-022-04449-2

**Published:** 2022-03-23

**Authors:** Hanan Fathy, Hamdi H. Hamama, Noha El-Wassefy, Salah H. Mahmoud

**Affiliations:** 1 Operative Dentistry Department, Faculty of Dentistry, Mansoura University, Algomhoria St, Mansoura City, 35516 Egypt; 2 Dental Biomaterials Science Dept, Faculty of Dentistry, Mansoura University, Mansoura, Egypt

**Keywords:** Resin-matrix ceramics, Hybrid ceramics, Ceramics, CAD/CAM, Indirect restorations

## Abstract

**Objective:**

To evaluate clinical performance of the new CAD/CAM resin-matrix ceramics and compare it with ceramic partial coverage restorations.

**Materials and methods:**

An electronic search of 3 databases (The National Library of Medicine (MEDLINE/PubMed), Scopus, and the Cochrane Central Register of Controlled Trials) was conducted. English clinical studies published between 2005 and September 2020 that evaluated the clinical performance of CAD/CAM resin-matrix ceramics inlays, onlays, or overlays were selected. The primary clinical question was applied according to PICOS strategy (Population, Intervention, Comparison, Outcome, Study design). The included studies were individually evaluated for risk of bias according to the modified Cochrane Collaboration tool criteria.

**Results:**

A total of 7 studies were included according to the established inclusion and exclusion criteria. From the included studies, 6 were randomized clinical trials while one study was longitudinal observational study without control group. According to the results of the included studies, the success rate of CAD/CAM resin-based composite ranged from 85.7 to 100% whereas the success rate reported for ceramic partial coverage restorations ranged from 93.3 to 100%. Fractures and debondings are found to be the most common cause of restorations failure.

**Conclusion:**

CAD/CAM resin-based composite can be considered a reliable material for partial coverage restorations with clinical performance similar to glass ceramic restorations. However, this result needs to be confirmed in long-term evaluations.

**Clinical relevance:**

CAD/CAM resin-based composites provide a potential alternative to ceramic indirect restorations. However, clinicians must be aware of the lake of knowledge regarding long-term outcome.

## Introduction

Preservation of biological tooth structures and obtaining excellent esthetic results are main goals of contemporary restorative dentistry [1, 2]. Restoration of large defects in posterior teeth, however, remains problematic among clinicians [3, 4]. The development of adhesive systems along with the existence of CAD/CAM technologies has made a conservative approach more applicable and capable of providing satisfactory results [2].

Conservative restorative dentistry has a wide range of minimally invasive techniques and systems for restoration of posterior teeth with large defects [4]. Indirect partial coverage restorations are considered to be a conservative substitute for crowns. They are used when the coronal part of the tooth is massively damaged and the remaining dentin thickness is too weak to support direct restoration [5]. They enable conservation of the remaining tooth structure and strengthen a compromised tooth with caries or a fracture [6–8]. According to the degree of destruction, partial coverage restorations can be classified into inlays (no cusp is covered), onlays (at least one cusp is uncovered), and overlays (covering all cusps) [6, 9].

Partial coverage restorations can be constructed using an array of materials such as ceramics, composites, and metallic alloys. Although metallic indirect restorations provide good mechanical results, patients have a desire for more tooth-colored materials. Therefore, ceramics and composites are materials of choice due to their excellent ability to match tooth colors [10].

Ceramic indirect restorations were first introduced in the late 1880s, but due to difficulties in construction and high failure rates, they did not become commonly used [11, 12]. The introduction of dental CAD/CAM technology and improvements in physical properties of ceramics have led to indirect restorations being fabricated from new materials such as leucite ceramics, lithium-silicate or lithium-disilicate ceramics, zirconia, and the newly developed resin-matrix ceramics [13]. These materials differ from each other in terms of their structure, composition, and properties.

Machinable ceramic blocks in a partially crystallized soft state allow milling without excessive diamond tool wear or damage to the ceramic material [14]. Leucite-reinforced glass ceramics are early generations of CAD/CAM blocks that were designed to increase the strength of feldspathic glass ceramics using the addition of leucite crystals up to 40% [15, 16]. They have been shown to have a favorable success rate of 95% after 5 years as partial coverage restoration [17].

Significantly higher strength was achieved by precipitating lithium-disilicate crystals in glass ceramics [15, 16]. Lithium-disilicate ceramics (LDCs) have a high crystalline content up to 70% and exhibit higher flexural strength when compared to leucite-reinforced glass ceramics [3]. CAD/CAM blocks are considered to be highly esthetic and these types of ceramic materials look best when glazed with oven firing. Therefore, these essential required additional steps can prolong chairside time procedures [18–21]. In order to shorten the chairside time, other lithium-disilicate strengthened lithium-aluminosilicate glass ceramics have been introduced and they do not require additional firing [19, 21, 22]. The addition of aluminum oxide also leads to improved strength [19, 21]. Hence, lithium-aluminosilicate ceramics (LAS) showed comparable flexural strength test results to LDCs, making it a high load-bearing material with excellent esthetic properties [22].

Zirconia is a crystalline dioxide of zirconium with mechanical properties similar to metals and a close-to-natural tooth color [23]. Compared to other ceramic materials, zirconia has superior strength, high fracture resistance, and excellent mechanical performance [23, 24]. The most challenging aspect of zirconia restorations is achieving a durable bond with the tooth structure due to its polycrystalline nature and it cannot be etched in the same way as other glass ceramics [25, 26]. The available studies have shown a 100% success rate for monolithic zirconia restorations as crowns after 36 to 68 months [27, 28]. In spite of this, the clinical evidence on partial coverage restorations made from zirconia is presently sparse.

A novel group of CAD/CAM materials has been recently introduced that have a composite resin-matrix and they are referred to as resin-matrix ceramics (RMCs) [16]. The rationale behind the introduction of this category of materials was to combine the advantages of polymers (low antagonist wear and improved flexural properties) and ceramics (color stability and structural durability) [16, 19, 29, 30]. The polymeric CAD/CAM materials are classified depending on their micro-structure and the industrial polymerization manufacturing mode for polymer-infiltrated ceramic networks (PICNs) and resin-based composites (RBCs) with dispersed fillers.

PICNs are characterized by a porous feldspar ceramic network that is infiltrated by a cross-linked polymer [16, 31]. Due to the dual network structure, the interlinked polymer network can mitigate crack propagation and improve the mechanical properties of the feldspar ceramic [32]. PICNs have demonstrated promising results in two studies with a 3-year survival rate of 97.0–97.4% for partial coverage restorations [33, 34].

CAD/CAM RBCs consist of a polymeric matrix reinforced by nano or nanohybrid ceramic fillers [31, 35]. This material has an elastic modulus close to dentin and has been demonstrated to have excellent flexural strength and internal discrepancy when compared to LDCs [31, 35]. However, these materials have their own limitations, such as discoloration, low fracture strength, and wear [2, 36]. Variations in mechanical properties between ceramic and resin-matrix ceramic materials raise questions about which material can survive longer in a load-bearing posterior region [6].

Many systematic reviews on the clinical performance of ceramic and indirect composite partial coverage restorations are available [6, 11, 22, 37–39]. However, systematic reviews that aim to evaluate the clinical success rate of CAD/CAM resin-matrix ceramics are still needed. In a review of composite versus ceramic partial coverage restorations, Fron Chabouis et al. [40] concluded that evidence to suggest the use of one material over the other is still limited. Therefore, this systematic review aimed to evaluate the clinical performance of CAD/CAM resin-matrix ceramic partial coverage restorations.

## Materials and methods

### Search strategy

The protocol of this systematic review was designed following the Preferred Reporting Items Systematic Review and Meta-Analysis (PRISMA) guidelines. The studies included in this systematic review were all clinical trials evaluating the clinical efficacy of CAD/CAM resin-matrix ceramics (RMCs) inlays, onlays, or overlays. The research question was as follows: Are CAD/CAM resin-matrix ceramics clinically efficient materials for partial coverage indirect restorations?

Initially, PICOS questions defined the search strategy as follows: P (population) patients who received the partial coverage restoration; I (intervention) included inlays, onlays, and overlays made of CAD/CAM RMCs or ceramics; C (comparison) between RMCs with ceramics; O (outcome) was the survival rate; and S (study type) randomized clinical trials and clinical follow-up studies. The keywords used were as follows: (“Computer-Aided design” AND “Composite resins” OR “Polymer-infiltrated ceramic” OR “Resin-matrix ceramics” AND “humans” OR “clinical trials” AND “Inlays” OR “Onlays”).

### Information sources

The following databases were searched for studies published between 2005 and 2020 that reported on the survival of resin-matrix ceramic partial coverage restorations: (The National Library of Medicine (MEDLINE/PubMed), Scopus, and the Cochrane Central Register of Controlled Trials). The studies were further checked manually.

### Study selection

Selection of studies went through three stages: (1) selection according to the title, (2) selection according to the abstract, and (3) analysis of the full text. All studies found by electronic and manual searches were collected and provided to each author. The eligibility criteria were checked by each author for all included studies. The agreement of at least two authors was required for the study to be selected.

### Eligibility criteria

The collected studies were assessed for the following inclusion criteria: clinical trials, related to CAD/CAM resin-matrix ceramic inlays, onlays, or overlays; studies written in English; and studies with clinical follow-up. After evaluating the studies according to the inclusion criteria, the following studies were excluded: studies published before 2005, non-English language manuscripts, protocols, case reports, and literature and systematic reviews. Laboratory studies and clinical studies that evaluated primary, abnormal, or endodontically treated teeth were also excluded. Moreover, direct composite and indirect restorations fabricated without CAD/CAM were excluded. In addition, clinical studies without follow-up or survival analysis were excluded.

### Data collection process

Data from the eligible studies were extracted by two reviewers (HF and HH) and collected into structured tables.

### Risk of bias of individual studies

The same authors (HF and HH) assessed the risk of bias in the selected studies using the modified Cochrane Collaboration tool, which included the following domains: selection bias (randomization, allocation concealment, unit of randomization issues), performance bias (blinding of participants, operators, examiners), detection bias (blinding of outcome assessment), attrition bias (loss to follow-up and missing values or participants), and reporting bias (unclear withdrawals or reported outcomes). We did not have access to study protocols so selective outcome reporting was not assessed in this study. Bias was assessed as a high, low, or unclear judgment. RevMan 5.4 (RevMan 5.4, The Nordic Cochrane Centre, The Cochrane Collaboration, Copenhagen, Denmark) was used to obtain a risk of bias summary and graph for the selected studies.

## Results

### Study selection

**Fig. 1:**
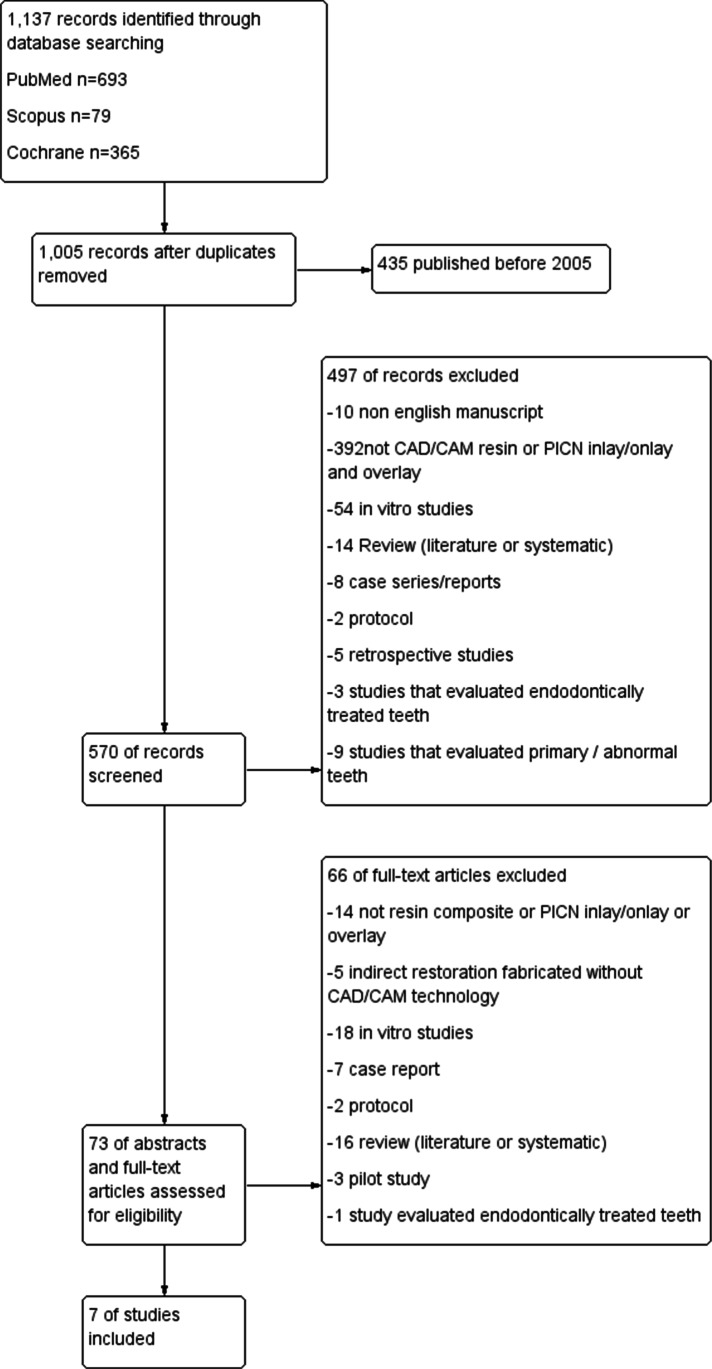
Study flow Chart

The search strategies identified 1,137 studies. After eliminating the duplicates and studies published before 2005, 570 studies were finally identified. A total of 497 of these studies were excluded after title evaluation. After evaluating 73 remaining studies, seven of the studies were included in this systematic review. The details of the selection process are illustrated in the flow chart shown in Fig. 1.

### Risk of bias assessment

**Fig. 2:**
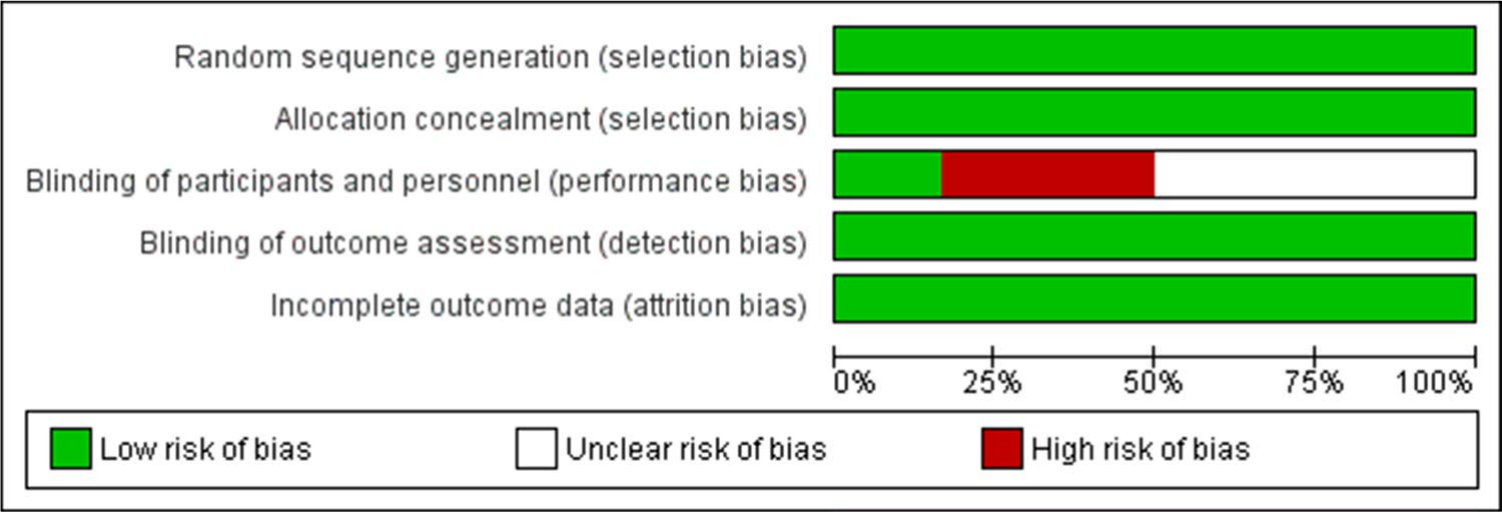
Risk of bias graph

**Fig. 3:**
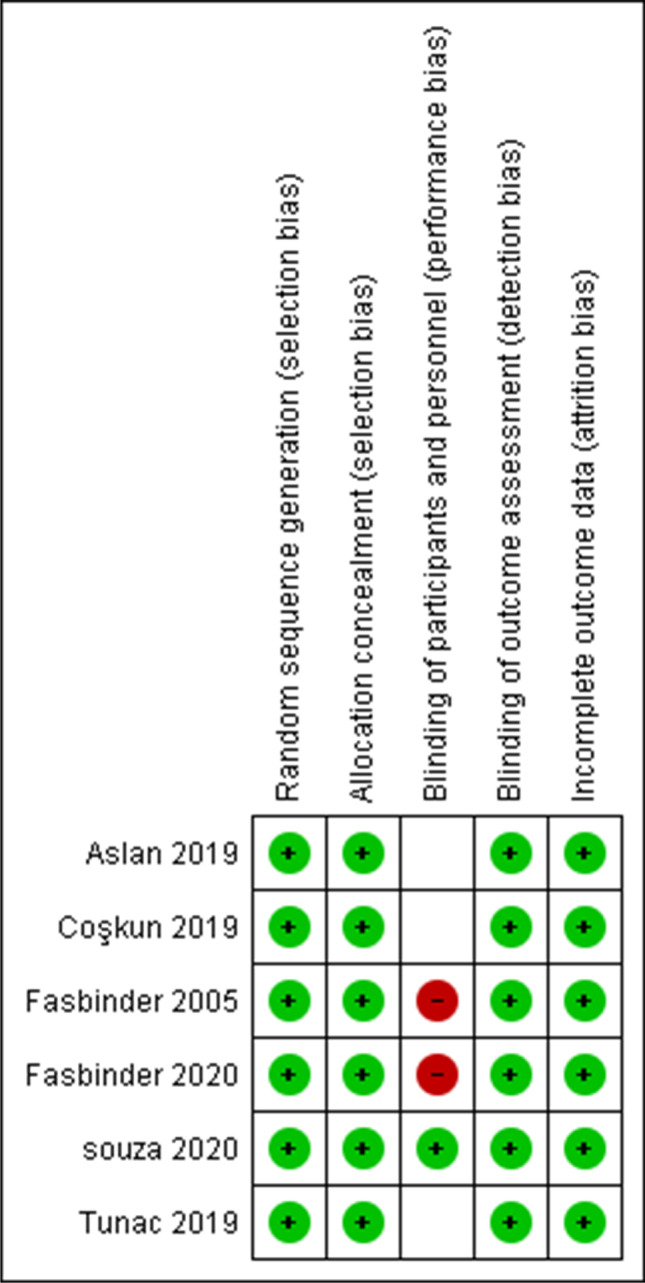
Risk of bias summary

A summary of the risk of bias assessment is listed in Table 1. The risk of bias was low overall for the remaining studies [3, 19, 30, 41–43]. Random sequence generation and allocation concealment were reported in all six studies. Blinding of participants and personnel was unclear in three studies [19, 30, 43]. It was obvious in these three studies that blinding of dental staff was not possible as the material can be recognized by an experienced eye, especially in the Tunac et al. study [43], which compared CAD/CAM resin composites with direct resin composites. After contacting the corresponding authors, it was ensured that blinding was not performed in Fasbinder et al. [41, 42]. In Souza et al. [3], the assessment was performed using a double-blinded design. In all six studies, clinical evaluations were performed by two independent examiners and the attrition bias was low. A risk of bias graph and summary are shown in Figs. 2 and 3, respectively.

**Table 1:** Risk of bias assessment summary

Study	Random sequence generation (selection bias)	Allocation concealment (selection bias)	Blinding of participant and personnel (performance bias)	Blinding of outcome assessment (detection bias)	Incomplete outcome data (attrition bias)
Souza et al. (2020)	Low	Low	Low	Low	Low
Fasbinder et al. (2020)	Low	Low	High	Low	Low
Coskun et al. (2019)	Low	Low	Unclear	Low	Low
Aslan et al. (2019)	Low	Low	Unclear	Low	Low
Tunac et al. (2019)	Low	Low	Unclear	Low	Low
Fasbinder et al. (2005)	Low	Low	High	Low	Low

### Study characteristics

The selected studies were published between 2005 and 2020. The material, objectives, and conclusions of each study are summarized in Table 2. The methodological assessment of the included studies included evaluation of the trial design, evaluation criteria, sample size, material selection, restoration type, surface treatment, isolation, cementation, follow-up, recall rate, and success (Table 3).

**Table 2:** Summary of studies included in the systematic review

Study	Year	Materials	Objective	Conclusion
Souza et al	2020	CAD/CAM resin-based composite (Lava Ultimate; 3 M) Lithium-disilicate glass ceramic (IPS e.max CAD ceramic; Ivoclar Vivadent)	To compare the 1-year clinical performance of lithium-disilicate and resin composite CAD/CAM onlay restorations	After 1 year of clinical service IPS e.max CAD and Lava Ultimate onlays showed a similar clinical performance that needs to be confirmed in long-term evaluations
Fasbinder et al	2020	leucite-reinforced ceramic (IPS EmpressCAD; Ivoclar Vivadent) nano-ceramic (Lava Ultimate; 3 M)	To measure the clinical performance of a nano-ceramic material (Lava Ultimate/3 M) for chairside Computer Assisted Design/Computer Assisted Machining (CAD/CAM) fabricated restorations	The nano-ceramic onlays had a lower incidence of fracture compared to the leucite-reinforced ceramic onlays with both having a very low risk of fracture. Nano-ceramic onlays performed equally as well as glass ceramic onlays over 5 years of clinical service
Coşkun et al	2019	Lithium-disilicate glass ceramic (IPS e.max CAD) Hybrid ceramic (Cerasmart, GC)	To evaluate the clinical performance of hybrid ceramic inlay-onlay restorations over a 2-year period	Based on the 2-year data, the tested hybrid ceramic can be considered a reliable material for inlay/onlay restorations
Aslan et al	2019	Lithium-aluminosilicate glass ceramic Lithium-disilicate glass ceramic CAD/CAM resin-based composite	To evaluate the clinical performance and the marginal adaptation of inlay/ onlay restorations made of lithium of a new lithium-disilicate strengthened, lithium-aluminosilicate glass ceramic (LAS) material compared with a conventional lithium-disilicate glass ceramic (LDS) and new-generation polymer-based CAD/CAM resin composite (CS) materials over one year	the CAD/CAM onlay restorations fabricated with lithium-disilicat-strengthened lithium-aluminosilicate glass ceramic (LAS) material showed similar clinical results and an acceptable level of marginal integrity to that obtained with lithium-disilicate glass–ceramic (LDS) materials and new-generation polymer-based materials; composite resin (CS)
Tunac et al	2019	CAD/CAM resin based composite (Lava Ultimate; 3 M) Direct nanohybrid resin Composite (Clearfil Majesty, Kuraray)	To evaluate the 2-year clinical performance of computer-aided design/computer-aided manufacturing (CAD/CAM) resin composite inlay restorations in comparison with direct resin composite restorations	Except the surface luster, 2-year clinical performance of CAD/CAM resin composite inlay restorations was found similar to direct resin composite restorations according to FDI criteria
Zimmermann et al	2018	CAD/CAM resin-based composite (Lava Ultimate; 3 M)	To describe initial clinical in vivo results for indirect particle-filled composite resin CAD/CAM restorations after 24 months	This study demonstrates particle-filled composite resin CAD/CAM restorations having a clinical success rate of 85.7% after 24 months. Adhesive bonding procedures need to be ensured carefully
Fasbinder et al	2005	Porcelain (Vita Mark II, Vita Zahnfabrik) CAD/CAM resin composite (Paradigm, 3 M ESPE)	To evaluate the longitudinal clinical performance of a resin-based composite (Paradigm, 3 M ESPE, St. Paul, Minn.) for computer-aided design/computer-aided manufacturing (CAD/CAM)–generated adhesive inlays	The resin-based composite inlays had a significantly better color match at three years than did the porcelain inlays. Resin-based composite CAD/CAM inlays performed as well as porcelain CAD/CAM inlays after 3 years of clinical service

**Table 3:** Methodological assessment

Study	Study design and evaluation criteria		Participant				Intervention				Comparison					Follow-up and recall rate		Outcome
	Trial design	Evaluation criteria	No. of patient	Age range (Y)	Gender	No. and type of teeth	No. and type of restoration	Surface treatment	Isolation	Adhesion protocol and cementation	G 1	G2		G3		Follow-up	Recall rate (%)	Success rate (%)
Souza et al., 2020	Split-mouth, RCT	FDI criteria	20	21–69 Mean age: 45 y	5 F, 15 M	Not mentioned	40 onlays 20 RBC, 20 LDC	RC: sandblasting (CoJet Sand; 3 M) + universal adhesive (Scotchbond Universal adhesive; 3 M) LDC: etching with 4.9% hydrofluoric acid (IPS Ceramic Etching Gel; Ivoclar Vivadent) + universal adhesive	Rubber dam	Selective etching of enamel for 30 s (Scotchbond universal etchant) + Universal adhesive (Scotchbond Univeral adhesive) self-etch resin cement (RelyX Ultimate, 3 M ESPE)	CAD/ CAM RBC	LDC		____		1 y (7 days, 1y)	100	100
Fasbinder et al., 2020	4 groups of two cements and two materials, RCT	Modified USPHS	86	Not mentioned	56 F, 30 M	38 premolars, 82 molars	120 onlays 60 leucite-reinforced ceramic, 60 nano-ceramic	Leucite-reinforced ceramic: etching with 4.9% hydrofluoric acid + silane coupling agent (Monobond Plus; Ivoclar) Nano-ceramics: air abrasion (CoJet Sand; 3 M) + universal adhesive (Scotchbond Universal adhesive; 3 M)	Isolite2 dryfield illuminator	1) Total etching for 20 s + A thin coating of Excite (Ivoclar) dentin bonding agent Total etch resin cement (Variolink II; Ivoclar) 2)Universal adhesive (Scotchbond Universal; 3 M) Self-etch resin cement (RelyX Ultimate; 3 M ESPE)	Nano-ceramic	Leucite-reinforced ceramic		____		5 y (baseline, 6mo, 1y, 2y, 3y, 5y)	100 (telephone interview)	_____
Coşkun et al., 2020	Split-mouth RCT	Modified USPHS	14	18–65	6 F, 8 M	10 premolars, 50 molars	60 (56 onlays, 4 inlays) (30 LDC, 30 HC)	Etching with 5% hydrofluoric acid (IPS Etching gel; Ivoclar Vivadent) for 60 s and 20 s for HC and LDC respectively + silane coupling agent (Monobond Plus; Ivoclar Vivadent) + Unfilled resin (Adhese Universal; Ivoclar Vivadent)	Rubber dam	Etching the enamel for 30 s and dentine for 15 s + Adhese Universal (Ivoclar Vivadent Total etch resin cement (Variolink Esthetic DC; Ivoclar Vivadent)	HC	LDC		____		2 y (6mo, 1y, 2y)	100	100
Aslan et al., 2019	3 parallel-groups, RCT	Modified USPHS	35	18–65	23 F, 12 M	15 premolars, 60 molars	75 (60 onlays, 15 inlays) (25 LAS, 25 LDC, 25 RBC)	Etching with 5% hydrofluoric acid gel (IPS Etching gel; Ivoclar Vivadent) for 20, 30 and 60 s for LDC, LAS and RC restorations respectively + ceramic primer conatianing silane coupling agent (Monobond Plus; Ivoclar Vivadent) + Unfilled resin (Adhese Universal; Ivoclar Vivadent)	Rubber dam	Etching the enamel for 30 s and dentine for 15 s + Adhese Universal (Ivoclar Vivadent) Total etch resin cement (Variolink Esthetic DC; Ivoclar Vivadent)	CAD/ CAM RBC	LDC		LAS		1 y (6mo, 1y)	100	100 (LAS and RC) 96.3 ( LDC)
Tunac et al., 2019	Split-mouth, RCT	FDI criteria	44	19–45 Mean age: 28 y	Not mentioned	65 premolars, 55 molars	120 60 indirect RC inlays, 60 class II direct composite	CAD/CAM RC: sandblasting (CoJet System, 3 M ESPE)	Rubber dam	For CAD/CAM RC: Three step etch and rinse adhesive system (OptiBond FL, Kerr) Self-etch resin cement (RelyX Ultimate, 3 M ESPE)	CAD/ CAM RBC	Direct RBC		____		2 y (6mo, 1y, 2y)	93.2	100
Zimmerman et al., 2018	Prospective observational study	Modified FDI criteria	30	56.4 ± 14.8	13 F, 17 M	13 premolars, 29 molars	42 RBC partial crowns	Air abrasion (CoJet; 3 M ESPE) + silane (Espe-Sil; 3 M ESPE)	Rubber dam	Etching the enamel for 30 s and dentin for 15 s then Syntac was used as adhesive bonding agent ( 15 s primer, 10 s adhesive) total etch resin cement (Variolink II; Ivoclar Vivadent)	Prospective observational study without control group					2 y (1y, 2y)	95.2 (after 12 months) 86.8 (after 24 months)	95 (after 12 months) 85.7 (after 24 months)
Fasbinder et al., 2005	2 parallel-groups RCT	Modified USPHS	43	Not mentioned	Not mentioned	43 premolars, 37 molars	80 inlays 40 porcelain, 40 RBC	Porcelain: Etching with 4.9% hydrofluoric acid + prehydrolyze silane coupling agent (RelyX Ceramic Primer; 3 M ESPE) RBC: air abrasion + universal adhesive (Single bond adhesive; 3 M)	Rubber dam	Total etching the cavity for 30 s + Single Bond (3 M; ESPE) total etch resin cement (RelyX adhesive resin cement (ARC); 3 M ESPE)	CAD/CAM RBC		Porcelain		___	3 y (6mo, 1y, 2y, 3y)	89	_____

Abbreviations:*RCT*, randomized clinical trial; *FDI*, World Dental Federation; *USPHS*, United States Public Health Service; *F*, female; *M*, male; *RBC*, resin-based composite; *LDC*, lithium-disilicate glass ceramic; *HC*, hybrid ceramic; *LAS*, lithium-aluminosilicate glass ceramic; *y*, year; *mo*, month

#### Evaluation of the trial design

Six of seven studies were randomized clinical trials (RCTs), and three of them were split-mouth studies [3, 30, 43]. The remaining study was a prospective observational study without a control group [44].

#### Evaluation of material selection

All seven included studies evaluated the clinical performance of CAD/CAM resin-based composites (RBCs). Two studies [33, 34] were found that evaluated the clinical performance of a polymer-infiltrated ceramic network (PICN) but they were excluded as they used endodontically treated teeth.

From the seven included studies, five compared RBCs with different types of ceramics. Two studies [3, 30] compared RBCs with lithium-disilicate glass ceramic (LDC). Aslan et al. [19] evaluated the same materials in addition to lithium-aluminosilicate glass ceramic (LAS). Fasbinder et al. [41] compared RBCs with leucite-reinforced glass ceramic. In another study [42] conducted by the same author, RBCs were compared with porcelain.

In the remaining two studies, Tunac et al. [43] compared CAD/CAM RBCs with direct composites. Zimmerman et al. [44] evaluated only RBCs with no control group.

#### Evaluation of restoration type (inlay/onlay or overlay)

The sample size and restoration types varied among the studies. Two studies [3, 41] only fabricated onlay restorations. Among them, Souza et al. [3] fabricated a total number of 40 onlays with half corresponding to each CAD/CAM restorative material used (20 LDC, 20 RBCs). Fasbinder et al. [41] fabricated a total number of 120 onlay restorations and divided them equally between leucite-reinforced glass ceramics and RBCs. In another study [42], 80 inlays were fabricated, half of them made of porcelain and the other half made of RBCs. On the other hand, two studies [19, 30] included both onlay and inlay restorations. Coşkun et al. [30] fabricated 60 CAD/CAM restorations, i.e., 56 onlays and 4 inlays, and the teeth were restored with 30 LDC blocks and 30 RBC blocks. Aslan et al. [19] fabricated 75 inlay/onlay restorations, i.e., 60 onlays and 15 inlays. The restorations were assigned to three groups, and each group involved 25 teeth restored with 25 restorations (LDC, LAS, and RBCs). In the study conducted by Tunac et al. [43], the fabrication of a total of 120 restorations was performed, 60 of them fabricated from CAD/CAM RBC blocks while the other 60 were class II direct composites. The last study conducted by Zimmerman et al. [44] included 42 RBC partial crowns but it was not clear whether the partial crowns were onlays or overlays.

#### Evaluation of the surface treatment method

In five studies [3, 41–44], air abrasion was used on the internal surface of RBCs. Four of these studies [3, 41, 43, 44] used 30-µm silica-coated particles (CoJet System; 3 M ESPE). Souza et al. [3] sandblasted the internal surface of lava ultimate restorations with CoJet Sand at 2 bar pressure for 20 s, while Tunac et al. [43] sandblasted it for 5 s. Fasbinder et al. [41] lightly air abraded the internal surfaces of the lava ultimate onlays with CoJet Sand in a micro-etcher. Finally, Zimmerman et al. [44] abraded the luting surfaces of the restorations with CoJet Sand (diameter 50 µm, 200 kPa). The four studies cleaned the restoration with alcohol followed by air-drying. Both Souza et al. [3] and Fasbinder et al. [41] applied Scotchbond Universal Adhesive (3 M) to the prepared internal surface of the onlays and then air-dried. Zimmerman et al. [44] applied silane (Espe-Sil; 3 M ESPE) to the restoration’s luting surface for a period of at least 60 s prior to adhesive luting.

Only one of the five studies [42] used air abrasion to the internal surfaces with 50-μm 40 pounds per square inch, and then, they applied a single layer of single bond adhesive (3 M ESPE) to the inlay and cured it for 20 s.

The remaining two studies [19, 30] etched the internal surface of the RBC restorations with 5% hydrofluoric acid (IPS Etching gel, Ivoclar Vivadent) for 60 s. Later, the restorations were rinsed and silanated with Monobond Plus (Ivoclar Vivadent). Then, the solvent was vaporized with compressed air. Unfilled resin (Adhese Universal; Ivoclar Vivadent) was applied to the internal surface of the restorations and dispersed with compressed air.

For ceramic restorations, the five studies that included LDC or leucite-reinforced glass ceramic restorations performed etching on the internal surface of the restoration with hydrofluoric acid (IPS Etching gel, Ivoclar Vivadent) for 20 s; then, they were sprayed with water for 20 s and air-dried [3, 19, 30, 41, 42]. For lithium-aluminosilicate glass ceramic, Aslan et al. [19] etched the restoration for 30 s.

Souza et al. [3] applied a universal adhesive after etching while Fasbinder et al. [41, 42] applied a silane coupling agent. Aslan et al. [19] and Coşkun et al. [30] silanated the restoration and then applied unfilled resin.

#### Evaluation of the isolation method

All the included studies utilized rubber dam as an isolation method except Fasbinder et al. [41] who used Isolite2 dryfield illuminator (Isolite > Zyris 6868A Cortona Drive; Santa Barbara, CA 93,117) in one of his studies.

#### Evaluation of cement type

All the included studies used resin cement for cementation of indirect restorations. Self-etch resin cement was used in two studies [3, 43] and total etch resin cement was used in four studies [19, 30, 42, 44]. One study evaluated the adhesive retention of CAD/CAM indirect restorations with a self-etch and total etch cementation technique [41].

#### Evaluation during the follow-up period and recall rate

The follow-up period was variable among all studies while the recall rate was almost 100% in the most of them. One-year follow-up was performed in Aslan et al. [19] and Souza et al. [3] which had a recall rate of 100%. Two-year follow-up was performed in three studies [30, 43, 44]. Among these three studies, the recall rate was 100% in Coşkun et al. [30], 93.2% in Tunac et al. [43], and 95.2% after 12 months and 86.8% after 24 months in Zimmerman et al. [44]. Three-year follow-up was performed in Fasbinder et al. [42] with 89% recall rate. Fasbinder et al. [41] followed the patients by telephone interview over a period of 5 years.

#### Evaluation criteria used in each study

FDI criteria were used in three studies [3, 43, 44]. The other four studies [19, 30, 41, 42] utilized modified USPHS criteria.

### Results of individual studies

#### Esthetic properties

##### Surface luster and finish

In Souza et al. [3], the luster of 80% of the lithium-disilicate ceramic restorations at baseline was rated as excellent, which was comparable to enamel, while 55% of CAD/CAM resin-based composite restorations were rated as clinically good with the rest rated as excellent or clinically satisfactory. After 1-year follow-up, the onlay restorations performed with both CAD/CAM materials exhibited deterioration in the surface luster without statistical significance for RBC restorations, but there was a significant difference in the case of LDC restorations.

Fasbinder et al. [41, 42], Coşkun et al. [30], and Aslan et al. [19] reported no significant differences in the surface luster between ceramic and RBC indirect restorations during different observation time periods. Fasbinder et al. [41] found that the surface of RBCs was comparable in smoothness and gloss to the leucite-reinforced ceramic restorations. Only by desiccating the surface of the restorations could the two materials be differentiated from each other, as the surface of the RBCs resulted in a matte appearance after desiccation.

Zimmerman et al. [44] reported that the surface gloss of CAD/CAM RBCs was stable with minimal surface abrasion after 12 months. However, after 24 months, the surface gloss deteriorated, but occlusal wear continued to be similar to that of enamel. Tunac et al. [43] reported no reduction in the surface luster of the RBC inlay group at the 2-year follow-up.

##### Color match

A study conducted by Souza et al. [3] showed that the translucency and color matches were considered excellent in approximately half of the restorations fabricated with either LDC or RBCs at baseline, as there was no difference in translucency or shade in comparison with the restored teeth. Esthetic deviation from the tooth was evident in the other half of CAD/CAM RBC restorations and in 20% of ceramic restorations. After 1 year, deterioration in the color match occurred in the onlay restorations performed with both CAD/CAM materials without a statistically significant difference from RBC restorations, but there was a significant difference in the case of LDC restorations.

Fasbinder et al. [41] reported that the USPHS criteria scores for color matching remained relatively unchanged over the 5-year follow-up period for both RBCs and leucite-reinforced ceramic. In another study, Fasbinder et al. [42] reported very good color matching for RBCs and porcelain restorations at baseline and it was maintained better by the RBC inlays at 3 years. Color matching of the porcelain inlays decreased at the 6-month follow-up, but then remained unchanged at the 3-year recall.

Coşkun et al. [30] and Aslan et al. [19] reported no significant difference in terms of color matching during recall evaluation for both RBCs and ceramic restorations. However, Aslan et al. [19] found that the translucency of LAS restorations was lower than that of restorations made of LDC and RBCs. Tunac et al. [43] and Zimmerman et al. [44] showed a decrease in color matching properties for the CAD/CAM resin composite at the 2-year follow-up.

##### Marginal staining

Minor marginal staining for both LDC and RBC restorations reported in Souza et al. after 1 year showed no difference between the materials [3]. Also, Fasbinder et al. [41] reported a very low incidence of marginal staining (3% of the onlays over 5 years) with no measured difference between RBC and leucite-reinforced glass ceramic restorations. In another study by Fasbinder et al. [42], there was no evidence of marginal discoloration at baseline and at 6 months for RBCs and porcelain. Also, there was no significant difference between the two materials at 3 years with 83.8% of RBCs and 91.2% of the porcelain inlays rated as alfa (no evidence of margin discoloration).

In Coşkun et al. [30] and Aslan et al. [19], all the restorations performed with RBCs or ceramics were rated as alfa at baseline. Also, there was no difference between the different materials at the 1- and 2-year follow-ups.

Tunac et al. [43] revealed that there was no reduction in marginal staining criteria after 2 years. On the other hand, Zimmerman et al. [44] reported deterioration in marginal staining in CAD/CAM RBC restorations at the 1- and 2-year follow-ups.

#### Functional properties

##### Fractures and debonding

There were no restoration fractures or debonding cases found in three studies [3, 30, 43]. Souza et al. [3] reported only chipping in the marginal bridge of one LDC case at the 1-year recall. Aslan et al. [19] reported that only one restoration from the LDC group exhibited minor fractures and there was no need for replacement, and only minor fractured surface polishing was performed.

Fasbinder et al. [41] reported that both CAD/CAM RBC and leucite-reinforced ceramic fractures were observed (four EmpressCAD and one lava ultimate) after 5 years of clinical service with no statistically significant difference between the materials. In the same study, a fracture of the adjacent tooth structure was observed with two lava ultimate onlays and an additional two onlays showed evidence of surface chipping that did not require treatment (one lava ultimate and one EmpressCAD).

Fasbinder et al. [42] rated three porcelain inlays as having a fracture but none of them required replacement; only one porcelain inlay was fractured at 3 years and was replaced with a porcelain onlay. None of the RBC inlays showed any evidence of fracture after 3 years. However, symptoms of incomplete tooth fractures developed in two RBC inlays and were restored with porcelain onlays.

Zimmerman et al. [44] reported the clinical failure of five CAD/CAM RBC restorations; three restorations failed due to debonding and two restorations failed due to cusp/tooth fractures.

##### Marginal adaptation

Souza et al. [3] found material adaptation criteria changed from excellent for 100% of the restorations to excellent for 90%, with two restorations being rated clinically good for both LDCs and RBCs after 1-year follow-up.

Fasbinder et al. study [41] showed increases in margin detectability for leucite-reinforced glass ceramic and RBC onlays with the margins of the RBC onlays being somewhat less detectable. However, all margins had alpha ratings. In another study [42], there was no significant difference between porcelain and RBC materials relative to marginal adaptation at 3 years. At baseline, 97.5% of the porcelain inlays and 100% of RBC inlays were rated Alfa-1 (undetectable with an explorer). After 3 years, 64.7% of the porcelain inlays and 62.1% of the RBC inlays were rated Alfa-1.

Coşkun et al. [30] reported that the marginal adaptation scores of both IPS e.max CAD and Cerasmart groups decreased from 100 to 96.7% with no statistical significant difference between them. Aslan et al. [19] found no statistically significant differences between the LDC, LAS, and RBC groups at baseline and after 1-year follow-up regarding the percentage of “continuous margin” at the total marginal length.

Tunac et al. [43] reported no reduction in marginal adaptation criterion for CAD/CAM resin composite inlays at the 1-year follow-up, and only slight deterioration was observed after 2 years without clinical significance. Zimmerman et al. [44] revealed a statistically significant difference for marginal adaptation criteria between baseline and the 2-year follow-up of lava ultimate restorations.

#### Biological properties

##### Postoperative hypersensitivity

In Souza et al. [3], patients reported minor postoperative hypersensitivity for five LDC restorations and for three restorations made with RBCs. One year later, this sensitivity was reduced with only one restoration from both materials rated as clinically good and not excellent.

In Fasbinder et al. [41], 10% of the patients reported slight sensitivity after 1 week but, after 4 weeks, all patients were asymptomatic without treatment. Fasbinder et al. [42] reported that only one inlay cemented with RelyX ARC had slight sensitivity at 1 week and sensitivity was resolved by the second week. There was no additional sensitivity reported in any of the inlays for both materials (RBCs and porcelain) at the 3-year recall.

In Aslan et al. [19], postoperative sensitivity was reported for the three materials (LDC, LAS, and RBCs) at baseline and it was resolved after 6 months. Zimmerman et al. [44] and Tunac et al. [43] reported no sensitivity during baseline and recall periods.

##### Recurrence of caries and tooth vitality

No secondary caries or endodontic complications were observed in any of the included studies.

##### Periodontal and mucosal responses

Improvement in periodontal response scores was reported in Souza et al. [3]. Improvement changed from 90% as excellent for both materials at baseline to 95% as excellent for LDC restorations and 100% for RBCs after 1 year. Also, Aslan et al. [19] reported that there were no statistically significant differences with plaque and gingival index values for LDC, LAS, and RBCs. In Tunac et al. [43], a slight decrease in periodontal response scores was reported after 2 years in the CAD/CAM RBC group.

In Coşkun et al. [30], there was no significant difference between the gingival index of the IPS.emax CAD and Cerasmart groups. The time-dependent gingival index values between baseline and 6 months, between baseline and 1 year, and between baseline and 2 years were significantly different in each group. The second year values were higher than the baseline values. A significant difference was observed in the plaque index of the Cerasmart group.

#### Success rate assessment

A 100% success rate was reported in three studies [3, 30, 43]. Aslan et al. [19] reported a low failure rate of 1.66% with a general success rate of 100% recorded for LAS and RBCs and 96.3% for the LDC group after 1 year. In Zimmerman et al. [44], the clinical success rate of indirect lava ultimate restorations was 95% after 12 months and 85.7% after 24 months. Fasbinder et al. [41] reported fractures in four (6.67%) leucite-reinforced glass ceramic resorations and three (5%) RBC restorations.

## Discussion

Systematic reviews are essential to collate the results reported in several studies and point out the best clinical evidence to support decisions clinicians make in their offices [45]. In the same context, systematic reviews decrease the time and expertise it would take to identify, locate, and appraise individual studies while adhering to proper scientific methodology [46].

The increased passion for conservation of the structure of teeth and advances in adhesive technology have increased the indications for partial coverage restorations [6, 47]. Indirect partial restorations are used in posterior teeth with excessive loss of dental hard tissues that cannot support a basic filling and as a conservative substitute for crowns [5]. High patient demand for more tooth-colored materials has supported the development of composites and ceramics with excellent tooth color matching the materials of choice [10]. Recently, CAD/CAM resin-matrix ceramic blocks were introduced; these materials aim to combine the positive advantages of polymers, which are not brittle, with ceramics, resulting in superior esthetics [16]. The search in this evidence-based study was dated back to 2005, representing the introduction date of hybrid ceramic restoration in the dental field. In light of the relatively new introduction of these types of restorations, the choice of material cannot be guided by esthetics alone but must also depend on clinical behavior [8]. Hence, the rationale for conducting this systematic review was to evaluate the clinical performance of CAD/CAM resin-matrix ceramic partial coverage restorations.

Clinical trials are very important in the practice of evidence-based medicine and health care reform. The impact of clinical trials extends not only to the individual patient by establishing a broader selection of effective therapies, but also to society as a whole by enhancing the value of health care [48]. A RCT is considered to be the gold standard of scientific human clinical investigation [49]. The two main features of RCTs are they are comparative studies and are designed to minimize bias [50].

The chance of partial indirect restorations failing in posterior teeth is higher for endodontically treated teeth as loss of vitality and excessive dentin removal affect the biomechanical behavior of teeth [6, 51]. These procedures as well may allow the passage of microorganisms and their by-products to the apical region of the root and into the alveolar bone, causing delayed failures [51, 52]. Therefore, endodontically treated teeth were excluded from this systematic review.

The restoration success rate is also influenced by several factors related to the patient, such as occlusal loads and caries risk [14, 53, 54]. Accordingly, a split-mouth design where the different restorations are randomly assigned to one of two-halves of the mouth removes much of the inter-subject variability, thereby increasing the power of the study compared to a parallel study [55].

Adequate adhesion is very important for the clinical success of restorations. Therefore, the cement type and a reliable surface treatment prior to cementation can enhance the bond strength and accordingly the long-term success of indirect restorations [56, 57]. Dual curing agents are preferred for cementation of indirect restorations to compensate for transmission of light throughout the restoration and to allow complete polymerization even at the bottom of the cavity, where access to LED curing light is limited [22, 58]. Bond strengths vary among specific cements, but total etch cements are generally still the system of choice due to their lower risk of hydrolytic degradation at the interface level and they provide the greatest retention as well [59, 60]. Cementation using resin cement is a very sensitive process; so adequate isolation is very important [59].

A reliable bond between the restorative material and luting agent is a critical factor that affects the long-term success of restorations [61]. Good adhesion to the internal surface of the restoration requires roughening of the internal surface of the restoration to increase the surface area for bonding, increasing the wettability of the cement to the restoration and forming chemical bonds between the ceramic, fillers, and cement. Restoration pre-treatment methods differ according to the material. The recommended method for conditioning the surfaces of ceramic restorations is to treat them with hydrofluoric acid and subsequently apply a silane coupling agent to ensure strong bonding [62–64]. For resin-matrix ceramics, chemical etching followed by silane application and alumina air abrasion followed by universal adhesive application are considered to be the best strategies for optimizing the bond strength of PICN material and RBCs under aged conditions, respectively [31].

The ability of CAD/CAM resin-matrix ceramic restorations to retain an esthetic and gloss surface over years of clinical service is questionable. The results of this systematic review showed that CAD/CAM resin-based composite restorations exhibited acceptable deterioration in esthetic properties (surface luster, color matching, and marginal staining) without statistical significance during different follow-up periods. Only Zimmerman et al. [44] reported significant decreases in surface gloss criterion after 24 months of clinical service. Their findings were in agreement with an in vitro report, as Koizumi et al. [65] reported that the surface luster and roughness of CAD/CAM RBCs could be altered by external manipulations such as tooth brushing.

The results also showed that regarding the esthetic criteria, there was no significant difference between CAD/CAM resin-based composite and ceramic restorations during different time periods, except for Fasbinder et al. [42] who reported that the resin-based composite inlays have significantly better color matches than the porcelain inlays at 3 years. The decrease in color matching for porcelain inlays was more a function of the modification of the tooth color over time rather than a discoloration of the porcelain itself. These results were consistent with the results of Molin et al. [66] in which a mismatch in color of the porcelain system increased from 15% at baseline to 50% at 5 years. Aslan et al. [19] as well found the translucency of LAS restorations was lower than that of LDC and RBCs. This opaque shade could have resulted from the alumina content added to increase strength.

Fractures and debonding are the major causes of restoration failures. The results of this systematic review showed better results for CAD/CAM resin-based composite partial restorations than for porcelain and leucite-reinforced glass ceramic restorations, but without clinical significance, and equal results when compared to lithium-disilicate and LAS glass ceramics. Lava ultimate has been reported to perform better under in vitro fatigue testing compared to several all ceramic materials due to a difference in their elastic properties [67]. It was reported to be less brittle and more flexible and had the best fatigue performance due to its greater resilience in enabling more stress absorption by deformation [67]. All ceramic materials had increased brittleness and cracking as the primary outcome [41]. However, not only the material characteristics but also occlusal forces play a relevant role in ceramic fractures [3, 68, 69]. Consequently, clinical reports have shown a higher failure risk in molars than in premolars [14, 69]. Also, they can originate from poor cavity preparation and improper cementation techniques [40, 61, 69–71]. This was in agreement with the findings of this systematic review and Zimmerman et al. [44] who reported that the tooth fractures observed in their study were related to a small cusp not being included in the preparation.

Failure due to debonding was found only in Zimmerman et al. [44]. This study showed for each debonding failure, the luting composite covered the tooth surface. Therefore, they assumed that the compound luting resin composite and restoration material might have been the weak point of the method. These results were in agreement with Frankenberger et al. [72] in which the laboratory bonding strength of different CAD/CAM materials using a micro-tensile bond strength approach was analyzed. The results showed the CAD/CAM RBCs have limited bond strength compared to ceramic materials and might be more susceptible to bonding failure if the bonding protocol and proper isolation are not carried out appropriately.

Marginal adaptation is also a critical factor in the longevity of indirect partial restoration. The increase in margin gap size may cause degradation in the adhesive bond resulting in microleakage and recurrent caries [42]. The use of suitable resin cement provides proper integration between the tooth and restoration, transferring the external forces to the dentin and improving the fracture resistance of the restoration [73]. Wear and ditching of resin cement have been reported in clinical evaluations of most ceramic onlays [14, 74–77]. In contrast, the interface between indirect resin composites and luting cement remained smooth without degradation due to similar mechanical properties [78]. This is in agreement with results of this systematic review, as increases in margin detectability occurred in CAD/CAM resin-based composite restorations at different time periods but without clinical significance. The results also showed no significant differences between ceramic and RBC restorations in marginal adaptation criteria, with the RBC restorations being less detectable.

Postoperative hypersensitivity was not a problem in all the included studies in this systematic review. A potential cause for minimizing postoperative sensitivity may be related to proper isolation during cementation and the use of a CAD/CAM technique with the ability to deliver the restoration in a single appointment, preventing tooth contamination during temporization and allowing bonding to occur to a freshly prepared tooth structure [79, 80]. Also, the use of manufactured blocks decreased polymerization shrinkage since it was limited to the resin cement thickness [81].

No recurrent caries or endodontic complications were observed in any of the included studies. Also, there were no significant differences between CAD/CAM RBCs and ceramics in terms of gingival and periodontal responses. Finally, the success rate of CAD/CAM RBCs was high overall. This material can be considered a new promising material that should be investigated further.

At the review level, our study contains a few limitations such as the exclusion of non-English manuscripts and variations in methodologies among the studies. Only a few studies were available that examined the clinical performance of CAD/CAM RBCs. Also, no studies were found that evaluated the clinical performance of polymer-infiltrated ceramic partial coverage restorations on vital teeth. The chance that eligible studies were not identified is low, as we searched three major databases and the reference lists of the included studies were checked. The results of this review relied on the reported results of previous studies and we preferred to analyze the original data, but some of the corresponding authors provided no information. Finally, evaluation with a longer follow-up period is still needed.

## Conclusion

CAD/CAM resin-based composite can be considered a reliable material for partial coverage restorations with clinical performance similar to glass ceramic restorations. However, these results need to be confirmed in long-term evaluations.
